# Retargeting FX-binding-ablated HAdV-5 to vascular cells by inclusion of the RGD-4C peptide in hexon hypervariable region 7 and the HI loop

**DOI:** 10.1099/jgv.0.000505

**Published:** 2016-08

**Authors:** Stacy Robertson, Alan L. Parker, Carolyn Clarke, Margaret R. Duffy, Raul Alba, Stuart A. Nicklin, Andrew H. Baker

**Affiliations:** ^1^​ British Heart Foundation Glasgow Cardiovascular Research Centre, University of Glasgow, Glasgow, UK

**Keywords:** gene therapy, adenovirus, coagulation factor X, targeting, hexon, fibre

## Abstract

Recent studies have generated interest in the function of human adenovirus serotype 5 (HAdV-5) hexon:  factor X (FX) binding and subsequent hepatocyte transduction and interaction with the immune system. Here, we retargeted adenovirus serotype 5 vectors, ablated for FX interaction, by replacing amino acids in hexon HVR7 with RGD-4C or inserting the peptide into the fibre HI loop. These genetic modifications in the capsid were compatible with virus assembly, and could efficiently retarget transduction of the vector via the αvβ3/5 integrin-mediated pathway, but did not alter immune recognition by pre-existing human neutralizing anti-HAdV-5 antibodies or by natural antibodies in mouse serum. Thus, FX-binding-ablated HAdV-5 can be retargeted but remain sensitive to immune-mediated attack. These findings further refine HAdV-5-based vectors for human gene therapy and inform future vector development.

Adenoviral (Ad)-based vectors, particularly those based on human adenovirus serotype 5 (HAdV-5) are widely used clinically and experimentally. *In vitro,* and in local *in vivo *applications, HAdV-5 transduces cells via the human coxsackie and adenovirus receptor (hCAR) (Bergelson *et al.*, 1997; Tomko *et al.*, 1997). Our research has focussed on the use of adenoviral vectors as a tool for *ex vivo* manipulation of coronary artery bypass material to overexpress anti- proliferative genes (e.g. TIMP-3, p53) in coronary artery vascular smooth muscle cells (VSMCs), to prevent their migration, proliferation and formation of a neointimal lesion, and ultimately graft reocclusion and failure following grafting (George *et al.*, 2011). A significant limitation in this strategy is that VSMCs express a very low level of CAR and are thus refractory to transduction (Parker *et al.*, 2013), necessitating high input titres of HAdV-5 to achieve therapeutic levels of transgene expression. For systemic *in vivo *applications, HAdV-5 efficiently and selectively transduces hepatocytes (Huard *et al.*, 1995) in a process mediated through the engagement of the blood coagulation factor X ( FX) with the hypervariable regions (HVRs) of the HAdV-5 hexon protein (Hofherr *et al.*, 2008; Kalyuzhniy *et al.*, 2008; Waddington *et al.*, 2008). Through selective modification of the HVRs, we previously generated a vector (HAdV-5T*) devoid of FX interactions and consequently hepatocyte transduction by introducing point mutations in key FX-interacting amino acids of the hexon protein HVR, namely T270P and E271G (HVR5) and I421G, T423N, E454S, L426Y and E451Q (all in HVR7) (Alba *et al.*, 2009). Conversely, it has been reported that FX may actually offer a protective role in gene delivery, by shielding HAdV-5 from immune-mediated attack by natural IgM and the classical complement system (Xu *et al.*, 2013), which interact with HVRs, neutralizing the virus (Ma *et al.*, 2015). The specific amino acids responsible for immune recognition remain unknown, and the impact in humans remains unconfirmed. Incorporation of FX-binding HVRs from HAdV-5 into HAdV-26 (non-FX-binding Ad serotype) instilled liver transduction to this vector (Ma *et al.*, 2015), reiterating the importance of FX in determining viral hepatic transduction. Therefore, retargeting of FX-ablated virus remains an area of interest for improving safety and efficacy of gene therapy vectors.

In this study, we evaluate optimal locations compatible for inserting targeting peptides within the HAdV-5T* vector. Previous studies identified the fibre HI loop as viable for peptide incorporation for retargeting *in vitro* and *in vivo* (Krasnykh *et al.*, 1998; Dmitriev *et al.*, 1998; Reynolds *et al.*, 1999). Furthermore, the incorporation of RGD into HAdV-5 HVR5 was shown to result in increased transduction in non-permissive VSMCs using a non-modified HAdV- 5 vector with high background hepatocyte transduction (Vigne *et al.*, 1999). Here, three locations were selected for peptide incorporation:  fibre HI loop (after aa 543G), and hexon HVRs 5 and 7 (Fig. 1a–d). We selected the RGD-4C (CDCRGDCFC) peptide to test retargeting because it efficiently binds to α_v_β_3_ and α_v_β_5_ integrins expressed on many cell types, including endothelial cells (Zitzmann *et al.*, 2002). This peptide has previously been widely used for adenoviral retargeting studies (Pasqualini *et al.*, 1997; Dmitriev *et al.*, 1998). The RGD-4C peptide was inserted into the HVRs with (designated ‘R’ for replacement) or without (designated ‘I’ for insertion) replacement of amino acids upstream of aa 272C (in HVR5) or 432K (in HVR7), whilst replacements involved removal of aa 272A–280L or 427T–435Q with simultaneous insertion of the peptide (Fig. 1c, d).

**Fig. 1.**
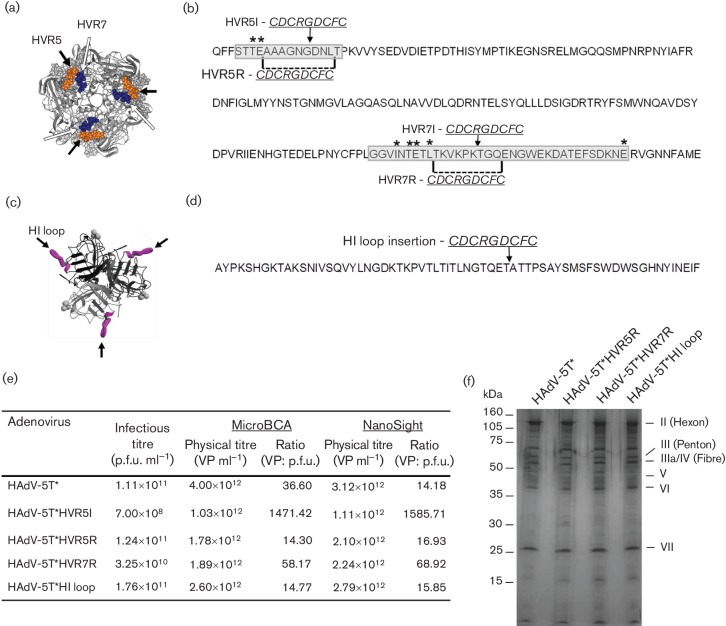
RGD-4C peptide placement in HAdV-5T* and quality control analysis of purified viruses. (a) Hexon trimer protein; arrows indicate RGD-4C insertion sites in HVR5 (orange) or HVR7R (blue). (b) HAdV-5 hexon amino acid sequence; HVR5 and HVR7 are highlighted in grey; T* point mutations are marked with an asterisks (*). The amino acids removed for generation of RGD-4C replacement vectors are designated by dashed lines and the RGD-4C insertion points are indicated by black arrows. (c) Fibre protein; arrows indicate RGD-4C insertion sites in HI loop (purple). (d) HAdV-5 fibre amino acid sequence; arrow indicates insertion site of RGD-4C. (e) Details of vector production, indicating preparation in HEK293 cells and infectious titres measured by end-point dilution infection in HEK293 cells, physical titres quantified by micro bicinchoninic acid assay (microBCA) and nanoparticle-tracking analysis (Nanosight) and the respective VP:  p.f.u. ratios for each virus. (f) Analysis of viral proteins by SDS-PAGE and silver-staining.

RGD-4C was cloned in modified shuttle plasmids containing the T* modified sequence (Fig. 1b) (Alba *et al.*, 2009) or fibre HI loop (Fig. 1d) (Alba *et al.*, 2010). Vectors were linearized and electroporated into BJ5183 bacteria cells with digested pAd5CMVlacZ for homologous recombination. Adenoviral production was performed in HEK293 cells as described previously (Alba *et al.*, 2009). Peptide insertion in HAdV-5T*HVR7I proved incompatible with virus assembly, suggesting limitations for peptide insertion within this locale. Virus generation could be achieved for HAdV-5T*HVR5I; however, titre assessment indicated very poor virus particle (VP):  p.f.u. ratios (Fig. 1e), again suggesting that simple insertion strategies within the HVRs appear to limit viral fitness, and this virus was therefore excluded from subsequent analysis. All other viruses were successfully propagated, and verified by sequencing, and quality control demonstrated consistent, high-quality virus batches, as assessed by silver-staining, bicinchoninic acid (BCA) assay, nanoparticle-tracking analysis (NanoSight LM10; Malvern) and p.f.u. assays (Fig. 1e, f), thus demonstrating amino acid removal from HVR5 and HVR7 is non-essential for virus assembly.

Recombinant viruses were evaluated for cell binding and transduction in α_v_-integrin-positive SKOV3 cells and A549 cells (Guo *et al.*, 2009; Cannistra *et al.*, 1995). Cells were transduced with HAdV-5, HAdV-5T* and peptide-modified adenoviral vectors at 50 p.f.u. cell^−1^, and transduction was assessed 48 h post-transduction after a 3 h exposure to each Ad by measuring β-galactosidase activity using the Tropix Galacton Plus and Tropix Accelerator II kit (Applied Biosystems). Replacement of amino acids in HVR5 with RGD-4C or its insertion in the HI loop failed to increase cell transduction compared with HAdV-5 or HAdV-5T*. However, replacing amino acids in HVR7 (HAdV-5T*HVR7R) with RGD-4C in the HAdV-5T* background exhibited >10-fold increase in transduction compared with the parental HAdV-5T* vector (Fig. 2a, b), validating HVR7 as a candidate site for targeting peptide insertion. Strategies to improve vascular tropism for effective gene delivery in coronary artery bypass graft purposes need to efficiently target VSMCs; therefore we tested viral transduction in low-passage (passage 2–5) human saphenous vein (HSV) primary VSMCs, isolated as previously described (Southgate & Newby, 1990). Using the RGD-targeted vectors (500 p.f.u. cell^−1^), we observed a robust sixfold increase in transduction in HSV VSMC transduced with HAdV-5T*HVR7R or HAdV-5T*HI loop compared with HAdV-5 or HAdV-5T*, but not HAdV-5T*HVR5R (Fig. 2c). This contrasts with previous studies demonstrating insertion of RGD in HVR5 in an otherwise WT HAdV-5 capsid, which did increase VSMC transduction (Vigne *et al.*, 1999). This discrepancy could relate to the different RGD peptide sequence used in each study; our peptide contained four cysteines (CDCRGDCFC) in contrast to only two in the previous study (DCRGDCF) (Vigne *et al.*, 1999). Additionally, these differences may relate to conformation alterations introduced by the specific combination of the FX-binding-ablating mutations engineered in HVR5 combined with the RGD peptide incorporation.

**Fig. 2.**
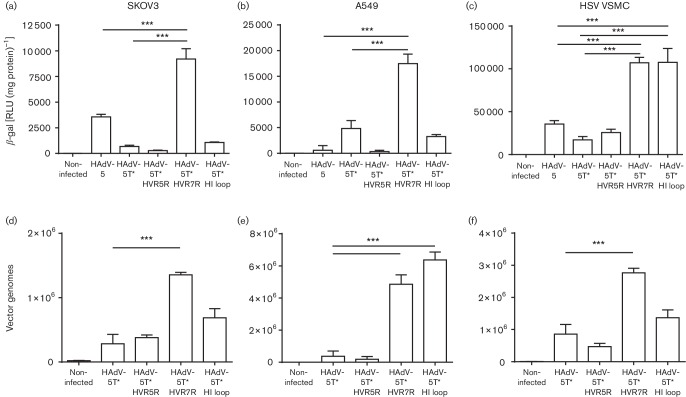
Assessment of HAdV-5T* retargeting by cell surface binding and viral transduction in three cell types. Cells were transduced with HAdV-5, HAdV-5T* and peptide-modified adenoviral vectors (HAdV-5T*HVR5R, HAdV-5T*HVR7R and HAdV-5T*HI loop) at a concentration of 50 p.f.u. cell^−1^ for SKOV3 and A549 cells and 500 p.f.u. cell^−1^ for HSV VSMCs. (a–c) Transduction was assessed 48 h post-transduction after a 3 h exposure to each HAdV, by measuring β-galactosidase activity. (d–f) Surface-binding analysis was performed by incubating the HAdV with the cells at 4 °C for 1 h and quantifying cell-bound adenoviral vectors by measuring adenoviral genomes by quantitative PCR as described in Alba *et al.* (2009). Each experiment was performed in technical triplicate and repeated three independent times. Mean±sd one-way ANOVA with Bonferroni post-hoc analysis was performed using GraphPad Prism v.5; ***P<0.001.

Surface binding analysis was performed by incubating the recombinant vectors with the cells at 4 °C for 1 h. DNA was then isolated using the QIAamp DNA Mini kit (QIAGEN) and cell-membrane associated viral genomes were quantified by quantitative PCR as described previously (Alba *et al.*, 2009). No change in binding was observed for HAdV-5T*HVR5R in any cell type, compared with HAdV-5T* (Fig. 2d, e, f), confirming that insertion of peptides within HVR5 confers little retargeting benefit, whilst significant increases in cell association were observed for HAdV-5T*HVR7R (Fig. 2d, f). Increased binding to A549 cells was observed following peptide insertion in the fibre HI loop, although this did not correlate with increased transgene expression (Fig. 2b, e). In HSV VSMCs, no increase in binding was observed for the HAdV-5T*HI loop; however, transduction was increased (Fig. 2f). This inconsistency could be due to differences in the ability of the virus to internalize and traffic through different cellular compartments following uptake. The discrepancy is observed across all of the cell types tested (SKOV3, A549 and HSV VSMC), indicating that the insertion of RGD-4C in the fibre HI loop may influence the surrounding capsid structure and hence affect trafficking to the nucleus. Further studies are required to fully delineate this finding.

FX binding HAdV-5 has been shown to prevent IgM- and complement-mediated neutralization of the virus *in vivo* (Xu *et al.*, 2013), with inhibitory serum components' binding sites within the HAdV-5 HVRs (1–3 and 5–7) (Ma *et al.*, 20 15). We tested if this response was altered by the insertion of the RGD-4C peptide within these regions by investigating the sensitivity of the vectors to murine serum *in vitro *using a snake venom-derived FX-binding protein (X-bp), which binds to the Gla domain of FX, preventing its interaction with the Ad hexon (Waddington *et al.*, 2008; Atoda *et al.*, 1998). Recombinant Ad vectors (2×10^10^ vpml^−1^) were incubated with RPMI-1640 medium, 90 % C57BL/6 mouse serum or 90 % C57BL/6 mouse serum pre-incubated with 40 µg ml^−^^1^ X-bp, for 30 min at 37 °C. Ad vector suspensions were diluted 200-fold in serum-free medium and 100 µl was added to SKOV3 cells for 2 h at 37 °C before being replaced with RPMI-1640 medium with 2 % FCS. Transgene expression was quantified 16 h post-transduction as relative light units (RLU) normalized to total protein. HAdV-5-mediated transduction significantly increased in the presence of serum, and reduced following pre-incubation of serum with X-bp (to bind and neutralize FX) (Waddington *et al.*, 2008; Mizuno *et al.*, 2001). HAdV-5T* demonstrated reduced transduction in the presence of murine serum compared with medium alone (Fig. 3a). Peptide insertion failed to prevent virus neutralization and reduction in transduction, indicating that these sites are not critical to natural antibody-mediated binding and neutralization (Fig. 3a). To evaluate what effect, if any, peptide insertion might have on evasion of pre-existing human anti-HAdV-5 immunity, we performed neutralization assays on HepG2 cell transduction following incubation with 1000 vp HAdV cell^−^^1^ in the presence of 1 IU ml^−1^ FX and in 2.5 % serum isolated from 103 cardiovascular patients (Parker *et al.*, 2009). Reporter gene expression was quantified 48 h post-transduction, and the changes in transduction relative to vector in the absence of serum were assessed (Fig. 3b). Incorporation of RGD-4C peptide into hexon or fibre had no discernible effect on evasion of pre-existing immunity, with 39.6 % evasion observed at the 90 % neutralization level for both HAdV-5T*HVR7R and HAdV-5T*HI loop compared with 35.9 % for the parental HAdV-5T* (Fig. 3b).

**Fig. 3.**
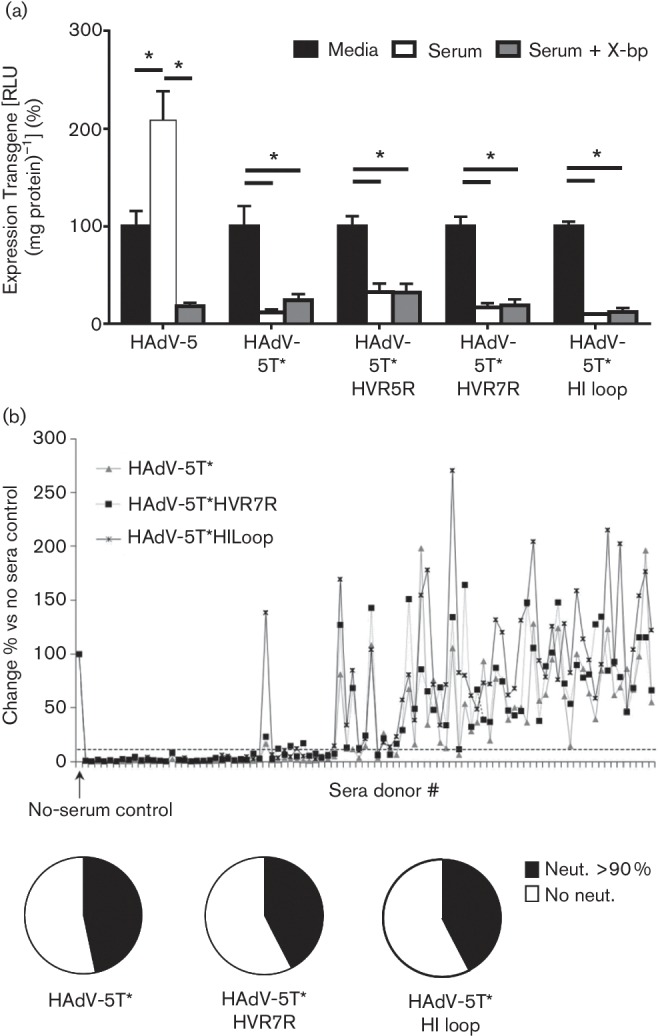
Evaluation of the effect of peptide insertion on evasion of neutralizing anti-HAdV-5 immunity. (a) HAdV-5T* and its derivatives (2×10^10^ vp ml^−1^) were incubated with RPMI-1640 medium, 90 % C57BL/6 mouse serum or 90 % C57BL/6 mouse serum pre-incubated with 40 µg ml^−1^ X-bp, for 30 min at 37 °C. Virus suspensions were diluted 200-fold in serum-free medium and 100 µl was added to SKOV3 cells for 2 h at 37 °C before being replaced with RPMI-1640 medium with 2 % FCS. Transgene expression was quantified 16 h post-transduction as RLU normalized to total protein. Transduction is expressed as a percentage of control (HAdV-5 transduction with serum-free medium alone); each experiment was performed in technical quadruplicate and repeated two independent times. Mean±se; *P<0.05. (b) Effect of neutralizing sera on HepG2 cell transduction following incubation with 1000 vp HAdV cell^−^^1^ vectors in the presence of 1 IU ml^−1^ FX and 2.5 % sera from patients previously screened for anti-HAdV-5 Nabs (Parker *et al.*, 2009) and stained for β-Gal expression 48 h post-transduction. The experiment was performed four times; data are presented as mean±se, and represent the percentage change relative to serum control.

This study successfully retargeted an FX-ablated HAdV-5 vector to human smooth muscle cells and demonstrated that incorporation of the RGD-4C-targeting peptide does not affect neutralization by natural antibodies in murine sera or recognition by pre-existing anti-HAdV-5 immunity in the general population. Whilst ablation of FX interactions increases neutralization of Ad via IgM and complement, this has only been demonstrated in murine models to date (Xu *et al.*, 2013). It remains unclear whether this is replicated in humans and this will be important to determine in the future. For intravascular delivery applications in humans, optimized retargeting strategies, including those based upon FX-binding-ablated Ad vectors described herein, will be of key importance. Further studies will be required to evaluate these vectors in an *ex vivo *human vein culture model (Soyombo *et al.*, 1990) and suitable *in vivo *animal models to confirm the ability of these modified viruses to target to vasculature using clinically relevant model systems.
